# A review of co-morbidity between infectious and chronic disease in Sub Saharan Africa: TB and Diabetes Mellitus, HIV and Metabolic Syndrome, and the impact of globalization

**DOI:** 10.1186/1744-8603-5-9

**Published:** 2009-09-14

**Authors:** Fiona Young, Julia A Critchley, Lucy K Johnstone, Nigel C Unwin

**Affiliations:** 1 Institute of Health and Society, 4th Floor William Leech Building, Medical School, University of Newcastle upon Tyne, Newcastle upon Tyne, NE2 4HH, UK

## Abstract

**Background:**

Africa is facing a rapidly growing chronic non-communicable disease burden whilst at the same time experiencing continual high rates of infectious disease. It is well known that some infections increase the risk of certain chronic diseases and the converse. With an increasing dual burden of disease in Sub Saharan Africa the associations between diseases and our understanding of them will become of increased public health importance.

**Aims:**

In this review we explore the relationships reported between tuberculosis and diabetes mellitus, human immunodeficiency virus, its treatment and metabolic risk. We aimed to address the important issues surrounding these associations within a Sub Saharan African setting and to describe the impact of globalization upon them.

**Findings:**

Diabetes has been associated with a 3-fold incident risk of tuberculosis and it is hypothesised that tuberculosis may also increase the risk of developing diabetes. During co-morbid presentation of tuberculosis and diabetes both tuberculosis and diabetes outcomes are reported to worsen. Antiretroviral therapy for HIV has been associated with an increased risk of developing metabolic syndrome and HIV has been linked with an increased risk of developing both diabetes and cardiovascular disease. Globalization is clearly related to an increased risk of diabetes and cardiovascular disease. It may be exerting other negative and positive impacts upon infectious and chronic non-communicable disease associations but at present reporting upon these is sparse.

**Conclusion:**

The impact of these co-morbidities in Sub Saharan Africa is likely to be large. An increasing prevalence of diabetes may hinder efforts at tuberculosis control, increasing the number of susceptible individuals in populations where tuberculosis is endemic, and making successful treatment harder. Roll out of anti-retroviral treatment coverage within Sub Saharan Africa is an essential response to the HIV epidemic however it is likely to lead to a growing number of individuals suffering adverse metabolic consequences. One of the impacts of globalization is to create environments that increase both diabetes and cardiovascular risk but further work is needed to elucidate other potential impacts. Research is also needed to develop effective approaches to reducing the frequency and health impact of the co-morbidities described here.

## Introduction

In Sub-Saharan Africa (SSA) infectious diseases still cause the majority of mortality (69% of deaths). Chronic non-communicable diseases such as cardiovascular disease, diabetes mellitus (DM), chronic respiratory disease and cancers, contribute around a quarter of deaths[1]. This picture is changing as SSA undergoes an epidemiological transition with a rapidly increasing burden of, and associated mortality from, chronic non-communicable diseases.

It has long been recognised that infective agents may predispose to, or trigger, some chronic non-communicable diseases with examples including infective contributions to cervical, liver and stomach cancers, and possible infective triggers for some types of diabetes[2,3]. In addition it is becoming clear that two of the most common infectious diseases in Africa, tuberculosis (TB) and human immunodeficiency virus (HIV)/acquired immune deficiency syndrome (AIDS), may also be closely related to chronic non-communicable diseases [4-13]. Diabetes predisposes to tuberculosis with some evidence that TB may also predispose to diabetes[10,14-16]. Antiretroviral therapy for HIV may increase the risk of metabolic syndrome (the clustering of abdominal obesity, hyperglycaemia, dyslipidaemia and hypertension) and thus predispose to type 2 diabetes and cardiovascular disease[6,7,9,11,17,18].

In this article we discuss the evidence on the relationships between TB and DM, and the possible mechanisms through which this link may be caused, we then discuss the link between antiretroviral therapy (ART), metabolic syndrome (MS) and cardiovascular disease (CVD). We also address the potential public health importance of these relationships within SSA and describe the possible impact of globalization upon these associations. In order to review these areas we have considered matters from biological, medical and social science perspectives where needed.

This article is based on detailed literature searches undertaken by the authors for articles published since 1950. Searches were undertaken in MEDLINE and EMBASE, plus screening of reference lists in identified articles. Potentially relevant reports, bulletins and guidelines were also screened, including ones from the United Nations (UN), World Health Organization (WHO), International Diabetes Federation (IDF) and UK Department of Health (DoH).

## Diabetes Mellitus (DM) and Tuberculosis (TB)

### Diabetes increases the risk of TB

An association between DM and TB has long been cited. In around 1000 A.D. Avicenna noted that 'phthisis', tuberculosis, often complicated diabetes[19]. In the UK, in the 1950's, some joint TB and diabetes clinics were set up to treat individuals with concomitant disease which were reported to improve outcomes[20,21]. However, until recent years there was a lack of good evidence on the strength and nature of the association between TB and DM. At present recognition of the association between diabetes and TB is low. The link goes unmentioned in many national and global TB control strategies even though it is plausible that diabetes is a major threat to effective TB control and the attainment of the TB Millennium Development Goals as well as other national and global targets [22-26].

Studies have shown an increased risk of TB infection in individuals with both type 1 and type 2 diabetes although measures of the strength of the association do vary[10,12,27-31]. Recently a meta-analysis of 3 studies carried out by Murray and Jeon showed that having diabetes was associated with a relative risk (RR) of 3.11 for contracting TB[10]. Stevenson *et al *carried out a systematic review of the subject finding that diabetes has been estimated to increase the risk of TB infection from 1.5 in one study up to 7.8 times in an other[12]. Stevenson *et al *also looked at the reported effects of age, gender and ethnicity upon the strength of the association between TB and DM. Reporting that gender did not seem to affect the RR for TB amongst individuals with DM, but that age did. The RR seems to be highest at younger ages and shows a decline as age increases, although this finding is not replicated in all studies, some show no association between the RR of TB amongst individuals with DM and of older age[12]. The increased risk of contracting TB for DM patients has been demonstrated in many different study populations. One study reports a difference in the association between TB and DM in different ethnic groups[28]. The study reports that DM is not a risk factor for TB in 'Black Hispanics' but is amongst 'White Americans' and 'Hispanics'. The cause of this finding is unclear; the investigators state that it may be due to unidentified HIV infection among 'black' controls which could attenuate any association found between diabetes and tuberculosis[28]. In order to gain a better awareness of the effect of ethnicity upon the strength of the RR of individuals with DM for contracting TB further investigation is needed.

Estimates of the population attributable risk (PAR) for TB from diabetes illustrate the potential importance of this relationship. PAR is dependent upon the prevalence of the risk factor (diabetes) and the strength of its relationship to the outcome (TB) and provides, given certain assumptions, an estimate of the proportion of the outcome that is directly caused by the risk factor. Stevenson *et al *estimated that diabetes accounts for approximately 14.8% of incident Pulmonary Tuberculosis (PTB) in India, and a slightly higher amount of TB infection has been found to be attributable to DM (25%) in a Mexican setting[16,30].

### Can TB increase the risk of diabetes?

Although the majority of studies identify and discuss the presence of diabetes as a risk factor for TB, the relationship between DM and TB is posited to be bidirectional. Early studies by Engelbach *et al* and Nichols *et al* reported that not only could having diabetes increase an individuals likelihood of developing TB but that having TB could lead to the presentation of diabetes[32,33]. Work carried out by Karachunskii *et al *showed that individuals with TB can develop changes in carbohydrate metabolism such as insulin deficiency and persistent hyperglycaemia[34]. Impaired glucose tolerance (IGT) and increased rates of DM have been found amongst TB patients in an African setting. A Tanzanian study found TB patients to have increased rates of IGT and a Nigerian study found an increased level of IGT and DM amongst TB patients. These results have been interpreted as an indication that TB can cause DM[14,15]. However, given the cross sectional rather than longitudinal design of these studies, their results are also compatible with undetected DM and IGT being present prior to the onset of TB. It is well known that in most populations a large proportion of those with DM or intermediate hyperglycaemia are undiagnosed and only detected upon blood glucose testing.

Although uncertainty remains around whether TB is a risk factor for DM it is clear that tuberculosis, as with other infections, complicates diabetes management and that some of the TB treatment regimes, including Isoniazid, have hyperglycaemic effects[35].

### Reported mechanisms causing the association

DM is known to impair immune function[36,37]. Specifically DM hinders cell mediated immunity and has been associated with low levels of leucocytes, polymorphonuclear neutrophils (PMNs) and a decreased T-helper1 cytokine response to TB [36,38,39] (PMNs produce cytokines and carry out phagocytosis[40]). T-helper 1 (Th1) type cytokines are vital in the control and inhibition of TB, for example, Interferon Gamma (IFN-γ) is important for combating microbial infection and both IFN-γ and Tumor Necrosis Factor alpha (TNFα, another Th1 cytokine) attack TB via the activation of macrophages [38-41]. Activated macrophages release reactive oxygen species (ROS) and free radicals such as Nitric Oxide which are essential for infection control, including TB infection[38,40]. Not only are macrophages the primary site of TB infection but they also instigate the main immune response to TB[12,41]. Macrophage function is inhibited in individuals with diabetes, the production of ROS, and phagocytic and chemotactic abilities are impaired. All of which are important for TB clearance[38,39,42]. Depressed immunity in DM patients would plausibly cause a higher risk of developing TB. Pulmonary microangiopathy occurs as a complication of DM and could partially explain the increased risk of lung infection for individuals with DM as well as the altered presentation of TB during co-morbidity[43]. Deficiencies in vitamins A, C and D are linked both with an increased risk of DM and TB. It has been hypothesised that there is a pathway upon which they act that dictates susceptibility to both diseases [44-47].

As afore mentioned some TB medications such as Isoniazid have been shown to have hyperglycaemic effects giving plausible mechanisms as to why glucose control is impaired in TB patients[35]. Inflammation caused by IL6 and TNFα whilst modulating a response to TB infection could cause an increase in insulin resistance thus decreasing insulin production causing an increase in blood glucose[48]. Although no singular mechanism has been identified as the cause of the associations between TB and DM many plausible causal pathways have been suggested.

### Effect on health outcomes when TB and DM are concomitant

An associated deterioration of both conditions occurs during co-morbidity with TB and DM. It is known that diabetes makes TB management more difficult and that chronic stimulation of the inflammatory system by TB may affect diabetes management and outcomes. In a study carried out in Mumbai, a higher mortality rate for tuberculosis when complicated with diabetes was seen. This increased mortality rate has been found elsewhere[49,50]. Co-presentation of TB and DM was associated with increased diabetes related complications in a recent study and poorer blood glucose control[13,28]. Tight blood glucose control is thought to reduce the risk of TB infection in an individual with DM[13,28].

Co-presentation with TB and DM has also been associated with more severe TB symptoms and clinical presentation; increased lung cavitations, and longer periods of smear positivity[51,52]. In co-morbid patients the involvement of the lower lung field is more common, Sosman and Steidl found multilobular cavitary TB was more common in people with diabetes[52]. However, there are relatively few studies that look at the lung pathology of co-infected DM-TB patients, data is sparse and sometimes contradictory, and as such it should be considered cautiously. In Nijmegen researchers monitored TB patients both with and without diabetes while they received treatment, they found that co-morbid individuals were more likely to have positive sputum results after six months of TB treatment, 22.2% compared with only 9.5% of the non-complicated TB cases. This suggests TB bacterial clearance takes a longer time in DM patients. Animal models have shown that hyperglycaemia causes higher TB bacterial loads than you would see in animals with normal blood glucose, this implies that infectivity is greater during hyperglycaemia which has implications for DM patients and could relate to the increased clearance times seen[53].

Data upon the effect co-morbidity has on the likelihood of the TB infection being multi-drug resistant is inconsistent. In a study carried out on the Texas border population it was found that multi-drug resistant TB (MDR-TB) was associated with DM with an Odds Ratio of 2.1[42]. Other studies have shown no increased association between DM and MDR-TB. Incompletion of TB treatment is a major cause of primary drug resistance. DM patients are thought to have impaired gastrointestinal drug absorption due to gastroparesis which may affect treatments. A study by Nijland *et al* reported that Rifampicin is not absorbed as effectively in TB-DM patients, this could again be due to poor gastrointestinal uptake, or due to differences in metabolism, excretion and body weight[54]. This poor intake of anti-TB drugs by DM patients could be a possible mechanism that leads to the development of drug resistance.

## HIV, Metabolic Syndrome, and Heart Disease

### Metabolic Syndrome

Metabolic abnormalities such as; glucose intolerance, Insulin resistance, abdominal adiposity high BP, and low HDL cholesterol and raised triglycerides tend to cluster, and it is the presence of these clustered abnormalities which are referred to as Metabolic Syndrome (MS). There is no universally accepted definition for MS but the 3 most often used are those as set out by the World Health Organisation (WHO), International Diabetes Federation (IDF) and National Cholesterol Education Program Adult Treatment Panel 3 (NCEPATPIII) [55-57]. Probably the most relevant to be used within an African setting due to its clinical accessibility is that of the IDF which requires for the diagnosis of MS central obesity, plus two of the following four additional factors: raised triglycerides, reduced high density lipoprotein cholesterol, raised blood pressure, or raised fasting plasma glucose[57].

### Anti Retroviral Treatment

Anti-retroviral treatment (ART) is the main management regimen for HIV/AIDS, it consists of a number of drugs that suppress viral replication and decrease viral load[58]. HAART (highly active antiretroviral therapy) is the gold standard for treatment where three or more drugs are combined in order to prevent the development of drug resistance. There are currently five classes of ART drug categorised on the basis by which they suppress HIV infection; Protease inhibitors (PIs), Nucleoside or nucleotide reverse transcriptase inhibitors (NRTI), Non nucleoside reverse transcriptase inhibitors (NNRTI), fusion inhibitors and integrase inhibitors[58]. Widespread use of ART in high-income countries has profoundly changed the outlook for HIV^+ ^individuals, reducing both morbidity and mortality. Once someone starts ART they will remain upon it for life.

### HIV and ART causing Metabolic Syndrome

The range of potential adverse consequences of ART is wide and includes gastro-intestinal disturbance, hepatotoxicity, pancreatitis, peripheral neuropathy, mitochondrial toxicity and anaemia[59]. Risk associations between HIV, its treatment, and various features of MS have been reported. It is during the treatment of HIV with ART that metabolic syndrome can be induced. The mechanism for this is unknown but it is thought to either be due to the infectious, inflammatory, process of HIV itself, a form of drug induced toxicity or perhaps through indirect effects. Two classes of ART, nucleoside reverse transcriptase inhibitors (NRTIs) and protease inhibitors (PIs) have been associated with inducing MS[18,60,61]. HIV treatment with protease inhibitors has not only been associated with hyperglycaemia, but the development of insulin resistance (a feature of MS and precursor to DM), increased levels of cholesterol and triglycerides, lipodystrophy, and the onset or complication of diabetes[18,60].

We will discuss further the association seen between HIV and three major components of MS, dyslipidemia, lipodystrophy and insulin resistance. Although these three features of MS are clearly inter-related the nature of these relationships are not yet fully understood, so, in order to describe their association with MS as noted in the literature we will do so separately.

### Risk of HIV Lipodystrophy (HIV-LD) in HIV^+ ^patients

HIV Lipodystrophy (HIV-LD) is seen in long term survivors of HIV infection, most of whom are receiving ART. HIV-LD is a complex syndrome thought to occur due to the secondary effects of HIV infection, direct drug-induced toxicities and, or, the indirect effects of changes in body composition on lipid metabolism[62]. The syndrome consists of both metabolic abnormalities (hyperlipidaemia and IR) and body fat redistribution (central adiposity and peripheral fat wasting). Central adiposity is manifest by the accumulation of visceral fat in the intra-abdominal space (abdominal obesity), dorsocervical spine (buffalo hump) and the breasts. Peripheral wasting describes loss of subcutaneous adipose tissue (lipoatrophy) in the limbs, face and buttocks in a generalised fashion. Both central adiposity and peripheral wasting can occur together but the underlying processes typically take place independently so that most often one feature is present alone[8]. The risk of central adiposity and peripheral wasting is greatly increased in HIV^+ ^patients on ART. In the Lancet, in 1997, the first report on body fat redistribution in an HIV^+ ^person associated with PI-treatment was published[60]. The following year, 1998, Carr *et al *designed a cross-sectional study to characterise the syndrome that was leading to this observed body fat redistribution and to determine if it was seen in association with all PI use or only in HIV patients using PIs. Healthy individuals, PI naïve HIV^+ ^patients and HIV^+ ^patients on PIs, were compared[18]. It was already known that Protease Inhibitors cause certain metabolic abnormalities such as hyperglycaemia but, this publication was the first to report that HIV patients on PIs had an increased risk of developing a syndrome of lipodystrophy with hyperlipidaemia and IR. It is now accepted that PI and other ART use in HIV^+ ^individuals are associated with fat redistribution. Studies on nevirapine [63] (an NNRTI) and stavudine, and lamivudine [59,64] (NRTIs) have all shown an association between usage and changes in fat deposition. All ART trials that have included objective body shape evaluation have consistently found an increased risk of abdominal fat in HIV patients regardless of which ART is used. However it is unknown which ARTs cause the most severe accumulation of visceral fat[65].

### Risk of Dyslipidemia in HIV^+ ^patients

Dyslipidaemia is characterised by hypertriglyceridaemia, hypercholesterolaemia and low serum high density lipoprotein (HDL) cholesterol, features of defective lipoprotein metabolism[6]. Although abnormal lipid profiles are reported in HIV^+ ^individuals before the onset of ART, hypertriglyceridaemia becomes both more prevalent and severe during treatment[66]. Sullivan *et al* in 1998 reported a case in which serum triglycerides markedly increased after 5 months of treatment with ritonavir (a PI). In the same patient there was also an increase in cholesterol, both concentrations returned to baseline 5 weeks after discontinuing ritonavir showing the association to be treatment rather than infection led[67].

Hypertriglyceridaemia and hypercholesterolaemia have been reported to occur with long term usage of drugs from the three main classes of ART, however, the association seems most common place with the use of PIs. Chen *et al *report prevalence of dyslipidaemia (defined as hypertriglyceridaemia, hypercholesterolaemia and low HDL) in HIV^+ ^individuals being treated with HAART as 70-80% and state that it can be associated with all available PIs[6]. It has also been reported that severe hypertriglyceridaemia associated with PI therapy can lead to acute pancreatitis[67].

### Risk of Insulin Resistance in HIV^+ ^patients

It is also known that HIV^+ ^people are at increased risk of IR due to the pro-inflammatory process of HIV, the direct effects of ARTs and also, indirect effects as consequences of ART (for example body fat distribution changes). The pathogenesis of ART-induced IR has been the focus of much discussion. Evidence suggests that body fat distribution changes cause increased fat deposition in muscle which is accompanied by impaired insulin sensitivity[17]. It has been shown that ART regimens impair glucose tolerance in one of two ways; induction of peripheral IR in skeletal muscle and adipose tissue and impairment of pancreatic beta cells to compensate[17]. It has also been reported that PIs bind to and block the insulin sensitive glucose transporter GLUT4[68]. Less is known about the mechanisms involved in the NRTIs effect on insulin sensitivity[11]. It has been well documented that IR is related to abdominal obesity, hypertriglyceridaemia and is associated with type 2 DM[18] There is much controversy as to whether it is changes in body composition that reflect underlying metabolic changes or vice versa[69]. In a recently published study in which ART-naïve patients were randomised to receive either an NRTI-regimen or an NRTI-sparing regimen, glucose and insulin were assessed before and approximately three months after initiation of therapy. The researchers reported that there was a reduction in peripheral insulin sensitivity without significant changes in body fat distribution in the NRTI group but not the NRTI-sparing group[70]. These findings indicate the changes are not mediated by alteration in body composition but that the risk is associated with NRTI usage.

### Risk of Heart Disease in HIV^+ ^patients

Magula and Mayosi (2003) looked at cardiac involvement in HIV patients and showed that abnormalities are commoner in HIV patients. Approximately half of hospitalised HIV patients and a high number of out-patients were found to develop cardiac abnormalities[71]. The DAD study (Data Collection on Adverse Events of Anti-HIV Drugs) assessed the risk of Myocardial Infarction (MI) in HIV patients by measuring the incidence of MI in terms of duration of HAART. The relative risk of an MI for an HIV patient on HAART was shown to be raised and to increase over time[7]. In another study cardiovascular disease risk was found to be significantly higher in HIV patients with MS in comparison to HIV patients with only abnormal body fat redistribution. This shows that MS increases the risk of MI more severely than body fat changes alone. Based on the Framingham criteria [72] the researchers report median percentage of cardiovascular disease risk at ten years for those with the MS and those without to be 10 and 5 respectively. It is not known how the traditional cardiovascular risk factors (e.g. smoking) modulate risk in the HIV population[66].

## Importance of these associations in a Sub-Saharan African setting and the impact of globalization upon them

### Importance of both associations within Sub Saharan Africa

Although much research is needed before we fully understand the biological pathways and effect on disease rates of the associations between the chronic and infectious diseases discussed in this paper it is clear that they could potentially have a large public health impact within Sub-Saharan Africa. In 2004 the WHO estimated there were 8.9 million new cases of tuberculosis, of which only half were reported to public-health authorities and, or, WHO[73]. The WHO African region has the highest estimated incident TB rate (356 per 100, 000 population per year) [73]. A large proportion of the increase in incident tuberculosis seen in Africa is attributable to the spread of HIV. In 2004 34% of newly diagnosed TB cases in Africa were estimated to be infected with HIV[73]. Diabetes Mellitus is a large cause of morbidity and mortality in Sub-Saharan Africa. The IDF has estimated that the prevalence of diabetes in SSA as a whole for 2006 was approximately 10.8 million, and they predict that this will rise by up to 80% by 2025 giving a prevalence of 18.7 million[74]. Could the large estimated rises in diabetes prevalence impact upon the future prevalence of TB, due to the association between the two diseases, as the rises in HIV have already been seen to?

As previously stated Stevenson *et al *estimated that Diabetes accounts for approximately 14.8% of incident Pulmonary Tuberculosis (PTB) in India, and a higher proportion of TB infection has been found to be attributable to DM (25%) in a Mexican setting[16,29,30]. These findings flag the potential public health importance of the association in Africa, although it must be noted that these estimates were not carried out within high HIV settings. As the numbers of individuals with DM rise it is plausible that there will be associated rises in the incidence of TB. The 2006 United Nations Joint Programme on HIV/AIDS (UNAIDS) report estimated that 63% (24.7 million) of all people infected with HIV worldwide resided in Sub-Saharan Africa and that the majority of deaths globally occurred here (72%, 2.8 million). All Southern African countries with the exception of Angola have an estimated adult HIV prevalence above 10%. In Botswana, Lesotho, Swaziland, and Zimbabwe, the estimated adult HIV prevalence exceeds 20%[75]. Effective treatment of HIV infection with antiretroviral therapy (ART) is now available even in countries with limited resources and in Africa the number of individuals receiving treatment has been greatly increased by the 3' by 5' campaign[75]. The large increase that has occurred in the number of people on ART has meant the number of people living with AIDS as a chronic condition has massively increased.

The WHO and UNAIDS 3' by 5' initiative, aimed to provide treatment to 3 million people in low and middle income countries by 2005. By December 2005, 18 countries had met their 3' by 5' target and 1.3 million individuals were receiving ART. In Sub-Saharan Africa, the number of people receiving HIV treatment increased more than eight-fold to 810,000 from 100,000. Despite these increases in ART, only 20% of those in need of treatment were receiving it by December 2005[75]. The G8 nations and the UN national assembly agreed to working with WHO and UNAIDS to continue developing an essential package of HIV prevention, treatment and care with the aim of moving as close as possible to universal access to treatment by 2010. The treatment of HIV with ARTs is a huge and greatly needed advance decreasing morbidity and mortality from HIV substantially but it has some unintended consequences that require either preventive efforts or appropriate treatment. If the goal of universal ART treatment within SSA is met then a substantial rise in metabolic syndrome, diabetes and heart disease may be seen. More research is needed to know how important this relationship will be globally and within SSA.

### The impact of globalization upon both associations within Sub-Saharan Africa and beyond

Globalization, which can be defined as a process in which regions are becoming increasingly interconnected via the growing movement of people, goods, capital and ideas has both positive and negative impacts on health[76]. One of the major processes indicative of globalization currently ongoing in SSA is urbanisation, resulting from a combination of natural population increase, reclassification of areas formerly considered rural, and rural to urban migration[77]. It is estimated that by 2020 the total urban population in SSA will double so that 487 million individuals will be living in urban areas. Growth of the urban populations within Sub Saharan African countries is occurring presently at an average rate of 4.5% per year[78]. Urbanisation in SSA, as in other less developed parts of the world, is strongly associated with increased levels of obesity, diabetes and cardiovascular disease[79]. In urban SSA obesity levels now equal those of the west[80]. Lower levels of physical activity [81] and an increasing calorie rich diet are key drivers of these increased rates. The production of processed foods has high profit margins and transnational food corporations are amongst the largest sources of foreign direct investment in many countries of Sub Saharan Africa[82]. Indeed, it has been appreciated for many years that the global availability and marketing of cheap vegetable oils and fats is leading to increasing fat consumption in less developed countries[83]. Obesity is the major risk factor for Type 2 diabetes, which accounts for over 90% of all diabetes[74], and rural-urban comparisons of diabetes in SSA find 2 to 5 fold higher prevalence in urban areas[81,84,85]. As an increase in diabetes prevalence occurs alongside rapid urbanisation, it is reasonable given the evidence reviewed here to suggest that this will make TB control more difficult, and may even lead to a rise in TB incidence. It is expected that numbers of individuals affected by the co-morbidities of diabetes and TB will rise. There are at least two million people who were born in Sub Saharan Africa now living in North America or Western Europe[86]. They have moved from a region of high risk for TB and HIV to countries with a lower risk of these conditions, meaning that they tend to be disproportionately represented in their host countries amongst those with TB and HIV. For example, according to the UK Health Protection Agency, there were over 1500 new cases of TB in the UK amongst people born in Africa in 2007, which is an annual rate of more than 300 per 100,000 compared to less than 10 per 100,000 in the white UK born population. In addition, people of African origin living in the UK, and other richer countries, tend to be at higher risk of diabetes, 2 to 4 fold higher, than the majority white population[74]. It is therefore highly plausible, but currently unknown, that international migrants from Africa to richer parts of the world are at much greater risk of the adverse combination of TB and diabetes. The potential importance of the relationships in international migrants, moving from Africa to richer parts of the world, is poorly researched and requires further attention. Labour migration patterns in Africa are considered one of the underlying determinants of the spread and distribution of HIV infection[87], which in turn is also linked to the spread of TB infection. Economic globalization is identified as one of the drivers of labour migration within Africa, both within and between countries, and particularly from rural to urban areas[88]. Thus an interaction is occurring between globalization, the risk of HIV infection and exposure to "obesogenic" urban environments. It is only with wider availability of ART that this combination becomes of public health importance giving a further increased risk to HIV positive people on ARTs of developing metabolic syndrome, diabetes and cardiovascular disease. Even with the afore mentioned side effects ART is the most essential response to the HIV/AIDs epidemic but the potential health effects of its prolonged use need to be addressed. Positive impacts of globalization are also seen. As the world becomes increasingly interconnected it has become easier to implement treatment in areas where disease is endemic and globalization, as represented through the activities of international organisations such as the United Nations/WHO, has played a large role in the increased access individuals within SSA now have to ART. Reduction in prices of ARTs for use within SSA by international drug corporations and the pledges from private donors have also contributed to this increased accessibility.

## Conclusion

SSA is currently seeing a very large change in the major health problems it faces. The link between chronic and infectious diseases becomes more important as the epidemiological transition in SSA progresses against a backdrop of globalization. In this review we reported upon the associations seen between two examples of chronic and infectious disease.

The literature reports a clear association between DM and TB and also discusses the possibility of this link being bi-directional. Although the underlying mechanisms for the association are not yet definite many possible pathways of action have been reported. The link between TB and DM will pose a serious threat to public health in SSA as DM prevalence rises. There are also published studies reporting an association between HIV, its treatment and many various features of metabolic syndrome. Although associations between HIV, its treatment using ART and HIV-LD, insulin resistance, dyslipidemia and heart disease are now accepted as occurring in western environments the mechanisms through which these occur are still under debate. More research is needed in low income countries in order to find the extent to which these issues will be a problem in SSA.

An awareness of the problems that occur due to the associations seen between chronic and infectious disease should allow us to deal with them more efficiently. More research however is needed upon the mechanisms of action for these risk associations in order for effective prevention or treatment of them to occur and more research needs to be carried out before we truly understand how globalization is impacting upon the associations.

## Abbreviations

AIDS: Acquired Immune Deficiency Syndrome; ART: Antiretroviral Therapy; CVD: Cardiovascular Disease; DAD study: Data Collection on Adverse Events of Anti-HIV Drugs study; DM: Diabetes Mellitus; HAART: Highly Active Antiretroviral Therapy; HIV: Human Immunodeficiency Virus; HIV-LD: HIV Lipodystrophy; IDF: International Diabetes Federation; IFNγ: Interferon Gamma; IGT: Impaired Glucose Tolerance; IL-4: Interleukin 4; IL-6: Interleukin 6; IL-12: Interleukin 12; MDR-TB: Multi-Drug Resistant TB; MI: Myocardial Infarction; MS: Metabolic Syndrome; NCEPATPIII: National Cholesterol Education Program Adult Trial Participants 3; NRTI: Nucleotide Reverse Transcriptase Inhibitor; NNRTI: Non-Nucleotide Reverse Transcriptase Inhibitor; PI: Protease Inhibitor; PMNs: Polymorphonuclear Neutrophils; PTB: Pulmonary TB; ROS: Reactive Oxygen Species; RR: Relative Risk; SSA: Sub-Saharan Africa; TB: Tuberculosisy; Th1: T-Cell helper one; TNFα: Tumor Necrosis Factor Alpha; UNAIDS: The United Nations Joint Programme on HIV/AIDS; WHO: World Health Organisation.

## Competing interests

The authors declare that they have no competing interests, that there are no conflicts of interest and/or financial disclosures where any of this work is identified. All authors have read and approved the final manuscript.

## Authors' contributions

FY and LJ were involved in the acquisition of data for this review. NU and JC were involved in the design of this review. FY drafted the review, LJ drafted the section on HIV, MS and CVD. NU critically revised the final review draft.
